# Antimicrobial nano-zinc oxide-2S albumin protein formulation significantly inhibits growth of “*Candidatus* Liberibacter asiaticus” in planta

**DOI:** 10.1371/journal.pone.0204702

**Published:** 2018-10-10

**Authors:** Dilip Kumar Ghosh, Sunil Kokane, Pranav Kumar, Ali Ozcan, Ashish Warghane, Manali Motghare, Swadeshmukul Santra, Ashwani Kumar Sharma

**Affiliations:** 1 Plant Virology Laboratory, ICAR-Central Citrus Research Institute, Nagpur, Maharashtra, India; 2 Department of Biotechnology, Indian Institute of Technology Roorkee, Roorkee, Uttarakhand, India; 3 Department of Chemistry, NanoScience Technology Center, University of Central Florida, Orlando, Florida, United States of America; 4 Department of Materials Science and Engineering, University of Central Florida, Orlando, Florida, United States of America; 5 Burnett School of Biomedical Sciences, University of Central Florida, Orlando, Florida, United States of America; Institute of Materials Science, GERMANY

## Abstract

Huanglongbing (HLB, also known as citrus greening) is considered to be the most devastating disease that has significantly damaged the citrus industry globally. HLB is caused by the *Candidatus* Liberibacter asiaticus (*C*Las), the fastidious phloem-restricted gram-negative bacterium, vectored by the asian citrus psyllid. To date, there is no effective control available against *C*Las. To alleviate the effects of HLB on the industry and protect citrus farmers, there is an urgent need to identify or develop inhibitor molecules to suppress or eradicate *C*Las from infected citrus plant. In this paper, we demonstrate for the first time an *in planta* efficacy of two antimicrobial compounds against *C*Las *viz*. 2S albumin (a plant based protein; ~12.5 kDa), Nano-Zinc Oxide (Nano-ZnO; ~ 4.0 nm diameter) and their combinations. Aqueous formulations of these compounds were trunk-injected to HLB affected Mosambi plants (*Citrus sinensis*) grafted on 3-year old rough lemon (*C*. *jambhiri*) rootstock with known *C*Las titer maintained inside an insect-free screen house. The effective concentration of 2S albumin (330 ppm) coupled with the Nano-ZnO (330 ppm) at 1:1 ratio was used. The dynamics of *C*Las pathogen load of treated Mosambi plants was assessed using TaqMan-qPCR assay every 30 days after treatment (DAT) and monitored till 120 days. We observed that 2S albumin-Nano-ZnO formulation performed the best among all the treatments decreasing *C*Las population by 96.2%, 97.6%, 95.6%, and 97% of the initial bacterial load (per 12.5 ng of genomic DNA) at 30, 60, 90, and 120 DAT, respectively. Our studies demonstrated the potency of 2S albumin-Nano-ZnO formulation as an antimicrobial treatment for suppressing *C*Las *in planta* and could potentially be developed as a novel anti *C*Las therapeutics to mitigate the HLB severity affecting the citrus industry worldwide.

## Introduction

Citrus greening disease (Huanglongbing, HLB) is economically the most serious and fatal disease of citrus throughout the globe [1, 2, 3, 4]. The disease was first reported in China in 1919 [1, 5] and has since spread all over the world. The causal agent of HLB is a fastidious, gram-negative bacterium, which exist in nature mainly in the form of three species, *viz*. ‘*Candidatus* Liberibacter asiaticus’ (*C*Las), [1, 6] ‘*Candidatus* Liberibacter africanus’ (*C*Laf) [7, 8] and ‘*Candidatus* Liberibacter americanus (*C*Lam) [9, 10]. The bacterium is phloem restricted and vectored by citrus psyllids [11, 12, 13]. Both *C*Las and *C*Lam are vectored by *Diaphorina citri* Kuwayama and ‘*Candidatus* Liberibacter africanus’ by *Trioza erytreae* [11, 14, 15]. The causal agent of HLB propagates in the phloem tissue of host and subsequent uneven distribution. The bacterium induces the deposition of callose and P-protein plugs throughout the phloem elements of infected plants. The deposition of plugs has been observed in both lateral pit fields as well as in and around sieve plates which causes numerous pockets of necrotic phloem. These plugs create blockage thus hindering the translocation of photoassimilates from source to sink tissue [16, 17, 18]. As a result photoassimilates convert to starch in all *C*Las affected living cells, eliciting yellow shoots, blotchy mottles on leaves, shoot die-back, off-tasting and malformed fruits, eventually contributing to an average yield loss of 30–100% [19, 1, 20, 21, 22]. It has been observed that as disease severity increases, the life span of infected citrus trees are reduced, and in severe cases the infected plants often die in 5 to 8 years [1, 23]. The *C*Las bacteria are inaccessible for most antimicrobial and bactericidal agents; therefore still there is a lack of efficient control strategy at commercial level. There are no known citrus commercial cultivars with natural resistance against HLB [1, 22].

There are limited control options available to the farmers that include elimination of psyllid vectors from the field using insecticides, reducing *C*Las inoculums by eradicating infected citrus plants from citrus groves and raising the HLB-free planting material in protected nurseries [1, 24]. The development of HLB tolerant citrus varieties is one of the most promising approaches, but unfortunately there is lack of HLB resistant genes [21, 25]. Some recent reports demonstrate reduction of *C*Las titer after treatment, i.e. graft-based chemotherapy, thermotherapy [26] and use of antibiotics *i*.*e*. penicillin, carbenicillin and streptomycin [27, 28, 29, 30, 31]. Several countries are trying to develop anti-HLB therapeutics involving injecting trees with different curative agents such as antimicrobials, bactericides and antibiotics to reduce the titer of *C*Las. However, the antibiotics treatment has adverse collateral effects *i*.*e*. appearance of antibiotic resistant bacteria and secondary delivery to consumers [32]. Development of inhibitor molecules against *C*Las and generation of transgenic cultivars, to over-express the inhibitor molecule, would be a potent approach to control or manage HLB. Hu et al. [33] reported eleven SecA inhibitors and developed a micro-emulsion formulation for their active delivery *in planta*. Recently, the HLB tolerant transgenic cultivars (Hamlin, Valencia) were developed, expressing NPR1 gene of *Arabidopsis thaliana* with a phloem specific SUC2 promoter [34]. The effective treatment with Brassinosteroids against HLB has also been reported. However, this remediation treatment has not been used at the commercial level yet [24].

Zinc Oxide has traditionally been used as a fertilizer in agriculture industry. The U.S. Food and Drug Administration (US FDA) listed ZnO as being generally recognized as safe (GRAS) if it is used as food additive. The U.S. Environmental Protection Agency (US EPA) has recently exempted ZnO (CAS Reg. No. 1314-13-2) from the requirement of tolerance when is used as an additive upto 15% (wt/wt) in bactericide/fungicide formulations [35]. Beneficial effect of Nano-ZnO (~10 nm) on cucumber fruit (*Cucumis sativus*) was reported in greenhouse settings [36]. As reported, soil application of Nano-ZnO through at 400 and 800 mg/kg rates did not demonstrate any negative effect on carbohydrate and antioxidant contents but increased starch and protein content in comparison to untreated controls. Furthermore, Nano-ZnO is more soluble than fertilizer-grade bulk ZnO due to increased surface area. Therefore, it is expected that Nano-ZnO will eventually degrade to Zn ions and become a part of micronutrient pool, contributing to productivity improvement. Other carbon based nanomaterials such as carbon nanotubes [37], nano-onions [38] are shown to contributing to productivity increase, presumably due to their activity as plant stimulant. Antimicrobial properties of ZnO also have been investigated. Several studies reported effective antibacterial activity of Nano-ZnO against gram-negative bacteria *Escherichia coli*, *Xanthomonas alfalfa*, *Pseudomonas aeruginosa*, and gram-positive bacteria *Staphylococcus aureus* [39, 40]. Detailed mechanisms of antimicrobial activity of Nano-ZnO have not been fully understood yet. Suggested mechanisms of killing are linked to release of antimicrobial zinc ions and production of reactive oxygen species [41, 42]. The use of antimicrobial proteins is one of the most recent advances in the field of agriculture molecular biology and nanotechnology towards plant disease control [43, 44, 45]. Several studies reported good antifungal, antibacterial and serine proteinase activity of 2S albumin family proteins. The ability of 2S albumin protein to inhibit microbial growth has been also reported and it is suggested as an important target protein for designing antifungal drugs [46]. The SiAMP2, a 2S albumin family protein from *Sesamum indicum* kernels, has been shown as detrimental to *Klebsiella* species [47]. Our preliminary experiments have indicated successful inhibition of *E*. *coli* (DH5α), a *C*Las family of gram negative bacterium, cultured *in vitro* using media containing varying concentrations of 2S albumin protein, extracted from the seeds of pumpkin (*Cucurbita maxima*). This suggests the possibility of applying 2S albumin protein as one of curative agents against HLB. The pumpkin 2S albumin is heterodimeric (~12.5 kDa in size) seed storage protein that is thermally stable and water-soluble. This protein has demonstrated anticancer, RNase / DNase and antifungal activities. Far-UV (190–260 nm) CD spectroscopy studies for secondary structure showed that the protein is thermally stable up to 90°C, retaining its alpha helical structure [48, 49].

In the present investigation, with the objective of developing effective antimicrobials against *C*Las, we evaluated the antimicrobial efficacy of a trunk-injectable formulation containing 2S albumin protein isolated from *Cucurbita maxima* and Nano-ZnO both individually and in combination. Growth of *C*Las bacterium (measured as changes in its titer) in infected 3 year old Mosambi plants (*Citrus sinensis*) grafted on rough lemon (*C*. *jambhiri*) root stock was monitored after treatment to determine HLB killing efficacy.

## Materials and methods

### Production of HLB-positive experimental plants

**Fig 1:**
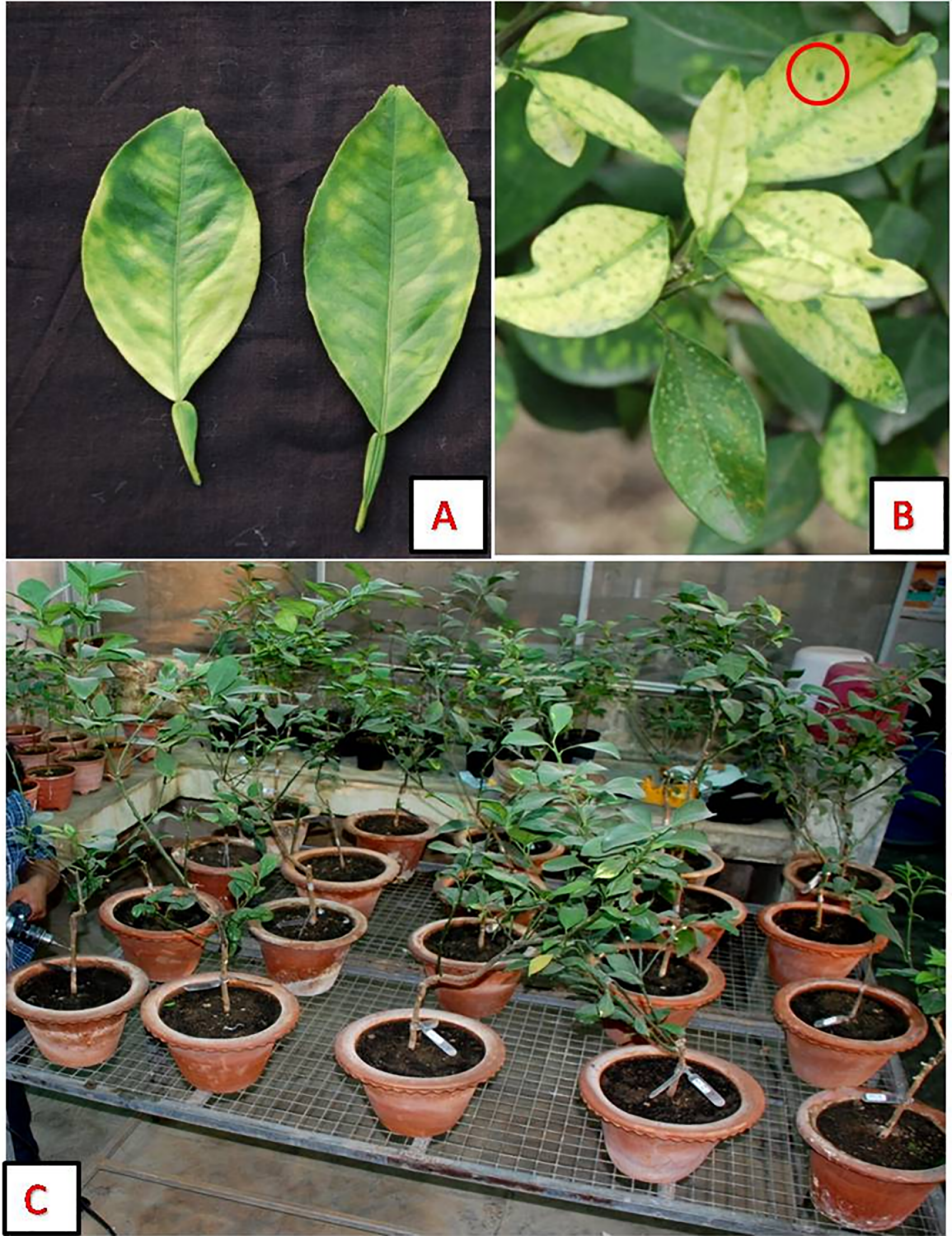
HLB disease symptoms on Mosambi (*Citrus sinensis*). (A) and (B) HLB symptoms of blotchy mottle and green island symptoms (Red circle) on leaves. (C) View of experimental HLB affected Mosambi plants.

Healthy two year-old Mosambi plants grafted on rough lemon rootstock were raised inside an insect-free screen house at ICAR-Central Citrus Research Institute, Nagpur, India. These plants were graft-inoculated with *C*Las-affected Mosambi scions and subsequently maintained in the screen house at temperatures ranging from 32±5°C (day time) to 22±5°C (night) during the entire period of the experiment. The plants showing typical HLB symptoms of blotchy mottle and vein corking on leaves after 3–5 months of graft inoculation (Fig 1A and 1B) were tested for *C*Las by conventional PCR using *C*Las specific primers (OI1/OI2c) [1] and PCR positive plants were selected for further investigations. Total of eighteen *C*Las positive and three healthy plants (Fig 1C) were selected based on positive conventional PCR. qPCR was performed for each of the experimental plants to determine the Ct values before and after the treatments as outlined below.

### Designing of primers, probe and TaqMan-qPCR assay conditions

The *C*Las specific primer pair (HLBas-F/-Rn) and probe (HLBp) based on sequences of 16S rDNA with an expected amplicons length of 76 bp (GenBank accession number L22532) were custom synthesized from Integrated DNA Technologies (Coralville, Iowa, USA) [50, 51]. HLBp (probe) was labeled at the 5’ terminal end with 6-carboxy-fluorescein (FAM) reporter dye and 3’ terminal end nucleotide with Black Hole Quencher (BHQ)-1dye (Table 1). This probe was used for standardization of TaqMan assay. An additional primer-probe set was synthesized on the basis of the sequence (GenBank accession number CX297817) of plant cytochrome oxidase (COX) [50]. The COX probe (COXp) was labelled with JOEN reporter dye with BHQ-2 at the 3’ terminal end and used as a positive internal control to assess the quality of DNA in reaction cocktails. The real-time PCR assay was performed using a Real Time PCR System (Applied Biosystems) in a total of 20 μl reaction volume consisting of the following reagents at the optimized concentrations: 300 nM (each) target primers (HLBas-F/HLBas-Rn), 200 nM target probe (HLBp), 300 nM (each) internal control primers (COXf and COXr), 200 nM internal control probe (COXp) with 1x TaqMan Universal Master Mix II (Applied Biosystems). The protocol was 95°C for 10 min, followed by 40 cycles at 95°C for 20 s, 58°C for 30 s and 60°C for 30 s. All reactions were performed in triplicate along with non-template controls.

**Table 1:** A list of primer and probe sets used for qPCR assay.

Sr. No.	Target gene	Primer/ Probe Code	Primer/Probe Sequence (5’- 3’)	Concentration (nM)	Amplicon Size (bp)	Reference
1	16S r DNA of *Candiadatus* Liberibacter asiaticus	HLBas-F	TCGAGCGCGTATGCAATACG	25	76	[50]
		HLBas-Rn	GCGTTATCCCGTAGAAAAAGGTAG	25		
		HLBp	56-FAM/AGACGGGTGAGTAACGCG/3BHQ_1	250		
2	Plant cytochrome oxidase	COX-F	GTATGCCACGTCGCATTCCAGA	25	68	
		COX-Rn	GCCAAAACTGCTAAGGGCATTC	25		
		COXp	56-JOEN/ATCCAGATGCTTACGCTGG/3BHQ_2	250		

### Specificity and sensitivity of TaqMan-qPCR assay

The specificity and sensitivity of the HLBas-F/Rn primers towards the target were evaluated. The sensitivity of assay and analysis of data were performed by StepOne Software v2.1 (Applied Biosystems). Standard curve and amplification efficiency was determined by use of 1:10 serially diluted genomic DNA (12.5 ng, 1.25 ng, 0.125 ng, 0.0125 ng, 1.25 pg, 0.125 pg and 12.5 fg) from *C*Las infected Mosambi plants.

### The synthesis of N-acetyl-L-cysteine (NAC) coated Nano-ZnO

The NAC coated Nano-ZnO was prepared using a wet chemical synthesis process. Zinc nitrate hexahydrate (1488 mg) (Zn(NO_3_)_2_.6H_2_O; Sigma-Aldrich; catalogue # 228737) was dissolved in 20 ml de-ionized (DI) water. Subsequently, 408 mg of NAC powder (Sigma-Aldrich; catalogue # A7250) was added and stirred until NAC was completely dissolved. Finally, the pH was adjusted to 9.0 with 1M NaOH.

### Purification of NAC coated Nano-ZnO

Excess NAC and other impurities present in the solution containing NAC coated Nano-ZnO product was removed by dialysis (Spectrum Labs, Spectra/Por 3 standard RC dry dialysis tubing, MWCO 3.5 kD). Dialysis was carried out for 72 hours changing DI water in every 8 hours. Nearly 300 mg of purified NAC coated Nano-ZnO powder was obtained after freeze-drying the entire dialyzed (initial volume of 25 ml) solution. Hereafter, the NAC coated Nano-ZnO was abbreviated as Nano-ZnO.

### Nano-ZnO characterization

High-resolution Transmission Electron Microscopy (HRTEM) was used to characterize particle size and crystalline phase of Nano-ZnO in vacuum state. Particle size and surface charge in solution were determined by Dynamic Light Scattering (DLS) using Malvern Zetasizer (model Nano ZS90).

### Determination of metallic Zinc content of Nano-ZnO powder

Atomic Absorption Spectroscopy (AAS; Perkin Elmar Analyst 400) was used to determine Zn content in Nano-ZnO. AAS sampling performed using 5 replicates for this study. In a 50 ml disposable centrifuge tube (VWR; Catalogue # 89039–658), 20 mg of Nano-ZnO powder was added to 20 ml of 1% HCl solution. The tubes were placed in a mechanical shaker for 12 hours to digest the particles. The resulting solution was clear and the pH was approximately 1.8 and showed no characteristic UV-Vis or fluorescence peaks, confirming complete digestion of Nano-ZnO. Metallic Zn content was determined to be about 53% (wt/wt).

### Phytotoxicity assessment of Nano-ZnO

Plant tissue damage potential (phytotoxicity) of Nano-ZnO and zinc nitrate were tested against ornamental vinca (*vincire*) plants (obtained from local Home Depot store) at 72 hours post foliar application. Ornamental vinca plants are susceptible to metal phytotoxicity such as Cu and used as industry standard. 3 plants were foliar sprayed with each treatments and controls at three different rates, 300 ppm, 600 ppm and 1,000 ppm. Test solutions were foliar applied using hand-operated pump mist sprayer till run off. Afterwards, plants were placed in plant growth chamber (Panasonic MLR-325H-PA) programmed to simulate summer conditions (maximum temperature set at 31°C). Visual observations were conducted after 72 hrs application to assess the overall plant health and phytotoxicity rating.

### Preparation of 2S albumin protein and Nano-ZnO formulation

2S albumin protein was isolated and purified as described earlier with some modifications from the seeds of pumpkin (*Cucurbita maxima*) [49] and the effective antimicrobial concentration was determined based on *in vitro* experimental data against *E*. *coli* (DH5α). Briefly, the pumpkin seeds were ground and soaked overnight in 50 mM of Tris-HCl. The extract was filtered and centrifuged at 18000 rpm for 60 min. The supernatant was passed through a pre-equilibrated DEAE-Sepharose column (1.5 × 10 cm) and the flow through was applied on to a CM-Sepharose column (1.5 × 8 cm) pre-equilibrated with 50 mM Tris-HCl buffer, pH 7.4. The bound proteins were eluted with a step gradient of NaCl in same buffer (50, 100, 300 and 500 mM). The fraction (eluted at 300 mM NaCl) was applied onto a gel-exclusion chromatography column (Superdex 75, 10/300 GL) pre-equilibrated with 50 mM Tris-HCl buffer, pH 7.4. The purity of the eluted protein was confirmed by a single band on a non-reducing 12% SDS-PAGE. The purified protein was concentrated to 0.33 mg/ml and used in further experiments.

### Trunk injection of 2S albumin protein, Nano-ZnO and Zinc nitrate

**Fig 2:**
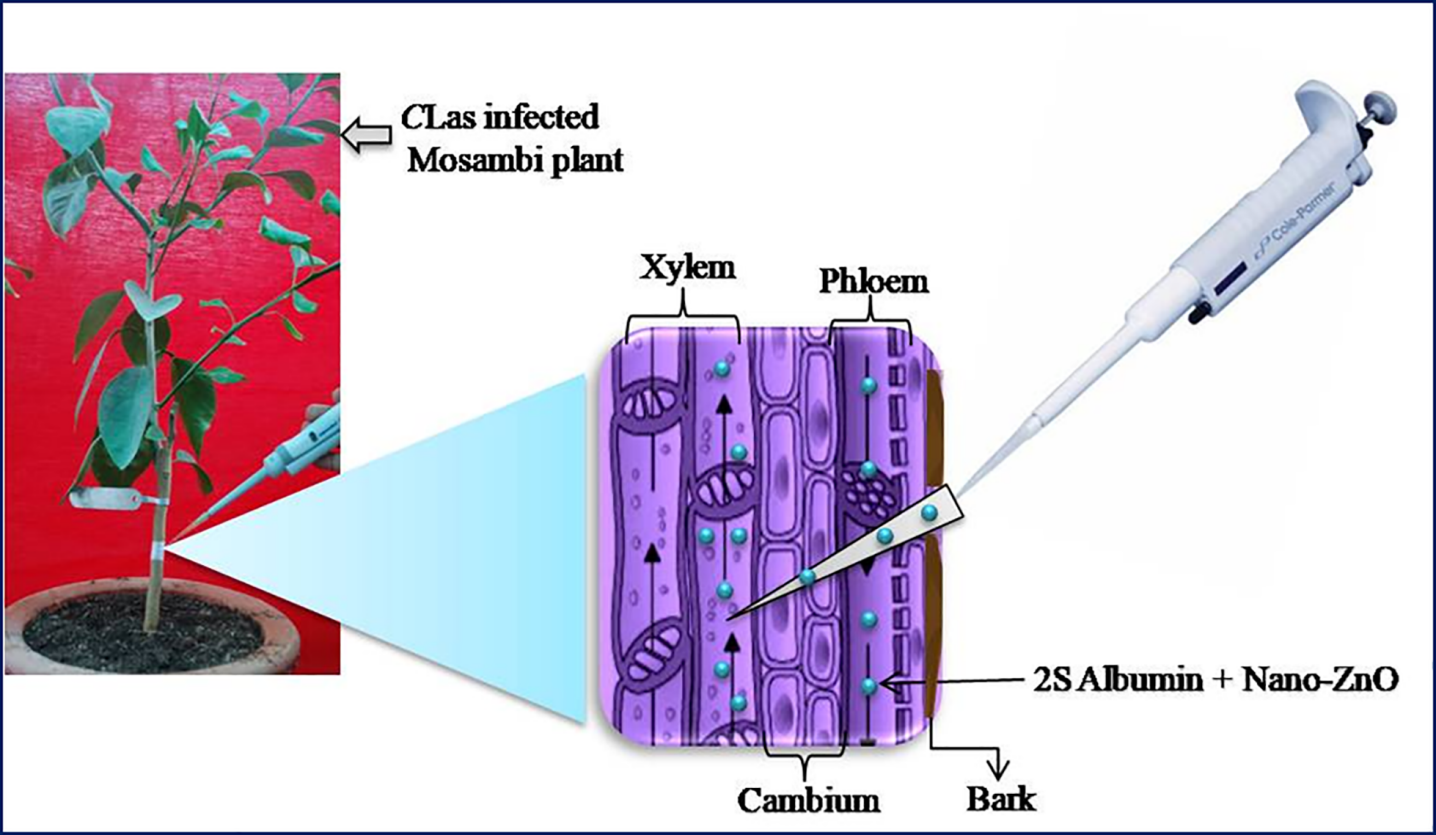
Schematic illustration of trunk injection of 2S Pumpkin seed albumin and Nano-ZnO into xylem-phloem region of Mosambi plants.

HLB infected, and healthy Mosambi plants with stem diameter of ~1.5 cm were injected with 2S albumin protein, Nano-ZnO and Zinc nitrate in five different combinations. A hole 2–3 mm diameter, and 6–8 mm depth was drilled on the trunk using a Kangaroo professional 10/13 MM IMPACT DRILL (Model No: KID 10/13 USA) with 2 mm bit. The drilled holes on trunk were 6 to 8 cm from the soil surface and at a 45° angle downward direction to avoid leakage of solution during trunk injection. The hole was drilled in such a manner that the end point of the bit would reach phloem and xylem region (Fig 2). The bits were surface wiped with 70% ethanol before and after use to prevent any secondary infection. Five different treatments were carried out: a) 2S albumin protein (330 ppm), b) Nano-ZnO (1,000 ppm), c) 2S albumin (330 ppm) plus Nano-ZnO (330 ppm), d) 2S albumin protein (330 ppm) plus Zinc nitrate (165 ppm) and e) Zinc nitrate (165 ppm). Each treatment had three replicates. Zn concentrations were carefully selected to minimize any potential phytotoxicity and residual effects. Preliminary phytotoxicity assessment was carried out against vinca model plants via foliar spray application, and it was found that Nano-ZnO was not phytotoxic upto 1,000 ppm (μg/mL) (S2 Fig). Zinc nitrate was found to be moderately phytotoxic even at 600 (μg/mL) and non-phytotoxic at 300 (μg/mL) (Table in S1 File). It was expected that the soluble form of Zn (such as Zn nitrate) would be at higher risk of causing phytotoxicity [52] than ZnO if it is trunk-injected at elevated concentration (e.g. 1,000 ppm metallic Zn). Three HLB infected drilled plants were taken as positive control (without any treatment) and three HLB-free plants were kept as negative control (healthy control). We used 330 ppm solution each of Nano-ZnO and 2S albumin protein individually in 10 mM PBS buffer (pH7.4). Desired amount of Nano-ZnO lyophilized powder was dissolved in PBS (pH 7.4), vortexed for 2 min and sonicated with Q SONICA sonicator (Ultrasonic Processor) for 5 min in glass vials. The prepared 2S albumin protein solution in PBS buffer was mixed in a 1:1 ratio with the Nano-ZnO solution and sonicated for 60 min. The plants having similar initial bacterial load were treated as outline above, for four months at monthly intervals from November 2016 to March 2017. The injected volume was 50 μl each day for three consecutive days of every month during morning hours. The holes were sealed with parafilm after injection to avoid leakage and opportunistic contamination. Same holes were re-drilled timely for subsequent timely injections.

### DNA extraction and evaluation of *C*Las load using TaqMan-qPCR assay

The experimental plant was injected with different combinations of treatment *viz*, a) 2S albumin protein, b) Nano-ZnO, c) 2S albumin plus Nano-ZnO, d) 2S albumin protein plus Zinc nitrate and e) Zinc nitrate and assayed over a period of four months. The *C*Las titer was determined in control and treated plants every 30 days. The collected leaves were washed with sterile water, wiped with 70% ethanol to avoid surface contamination and blot dried. Midribs and petioles were excised and ground in liquid nitrogen. And 100 mg of the sample was used for DNA extraction using the DNeasy Plant mini kit (Qiagen, Hilden, Germany) as per the manufacturer’s protocol.

### Monitoring of *C*Las titer in treated and control plants

The effectiveness of 2S albumin protein and Nano-ZnO against *C*Las load was analyzed by monitoring the titer of *C*Las using qPCR with TaqMan chemistry after every treatment before the first injection (0), and 30, 60, 90, 120 DAT. All treatments were performed in triplicate using negative controls (template from untreated healthy plant), positive controls (template from untreated HLB affected plant) and non-template controls. The data were analyzed using StepOne Software v2.1.

### Quantification of *C*Las genome copy number in experimental plants and data analysis

The quantification of *C*Las copy number was carried out using the standard curve method to determine the effectiveness of treatment. The fragment of 16S rDNA from *C*Las was amplified with the HLBas-F and HLBDr-3 primers pair (Table 2). The amplified PCR product (432bp) was cloned into the T-Vector pMD20 from Integrated DNA Technologies (Coralville, Iowa, USA). The cloned fragment was used as template for titer determination to generate the standard curve. The 10-fold serial dilutions in nuclease-free water were used (5.5 ng of initial template DNA up to 10^−9^). The initial concentration of the template DNA was estimated using Thermo Scientific NanoDrop™ 2000 Spectrophotometer. The standard curve was prepared with obtained Ct values using serially diluted template DNA by StepOne Software v2.1. The standard linear regression equation was obtained. The template DNA copy number was calculated with the help of following formula: Number of copies = (amount of target DNA in nano grams) x Avogadro’s number (6.0221 x 10^23^) **/** length of DNA amplicon in base pair (bp) x 660 x 1 x 10^9^. Finally, *C*Las copy number (per 12.5 ng of total genomic DNA) at 0, 30, 60, 90 and 120 DAT was calculated by extrapolating with the standard curve. We used equal quantity of DNA for the determination of *C*Las copy number after every month. Additionally, COX gene was used as internal control for normalization of the target gene.

**Table 2:** A list of primer set used for PCR product cloning.

Sr. No	Target gene	Primer Code	Primer Sequence (5’- 3’)	Concentration	Amplicon size (bp)
1	16S r DNA of *Candiadatus* Liberibacter asiaticus	HLBas-F	TCGAGCGCGTATGCAATACG	25nM	432
		HLB-Dr3	CTCGCCCCCTTCGTATTACC	25nM	

During evaluation of effectiveness of different treatments, the final genome copy number of *C*Las was calculated by subtracting the genome copy number at 30, 60, 90 and 120 DAT from initial genome copy number (at 0 days of treatment). The fold-change in *C*Las genome copy number was determined by dividing the average of total pathogen titer before the treatment by the total titer of each at 30 DAT. The effect of each treatment on *C*Las titre at different intervals were analysed using GraphPad Software. Significance differences were determined using two-sided paired t-test at a 95% confidence level. Statistical analysis revealed that variations in the *C*La*s* titre before and after treatment were significant (P < 0.05).

## Results

### Nano-Zinc oxide characterization

HRTEM measurements were carried out to determine Nano-ZnO particle size, size distribution and the crystalline phase of synthesized particles. Part a in S1 Fig shows representative HRTEM images of Nano-ZnO, showing particles in the size range 2.5 nm to 6 nm. HRTEM Selected Area Electron Diffraction (SAED) pattern revealed crystalling structures. The lattice spacing values were calculated from part b in S1 Fig and they were found to be 0.24 nm, 0.26 nm and 0.28 nm corresponding to [1 0 1], [0 0 2] and [1 0 0] lattice planes, respectively for ZnO Wurtzite (JCPDS card # 36–1451). HRTEM images were further analyzed to determine Nano-ZnO size distribution via processing multiple images obtained from the measurement (Part c in S1 Fig), and the average size was found to be 4.1 nm ± 0.7 nm. To further understand the dispersion properties of Nano-ZnO in water, hydrodynamic size were detremined via Dynamic Light Scattering (DLS) measurments (Part d in S1 Fig). The average hydrodynamic size was found to be 20.5 ± 6.7 nm (PDI: 0.129). The role of NAC coating was investigated via zeta potential measurements and zeta-potential value (ζ) -20.0 ± 7.1 mV at pH 7.50 (Part e in S1 Fig) was found. Negative zeta potential value is indicative of Nano-ZnO surafce coating with NAC and the presence of negatively charged carboxyl group on the particle surface. Presence of larger size particles in the solution state as measured by the DLS is indicative of particle-particle interactions causing slight particle agglomeration at nearly neutral pH conditions.

### Efficiency and sensitivity of TaqMan-qPCR

**Fig 3:**
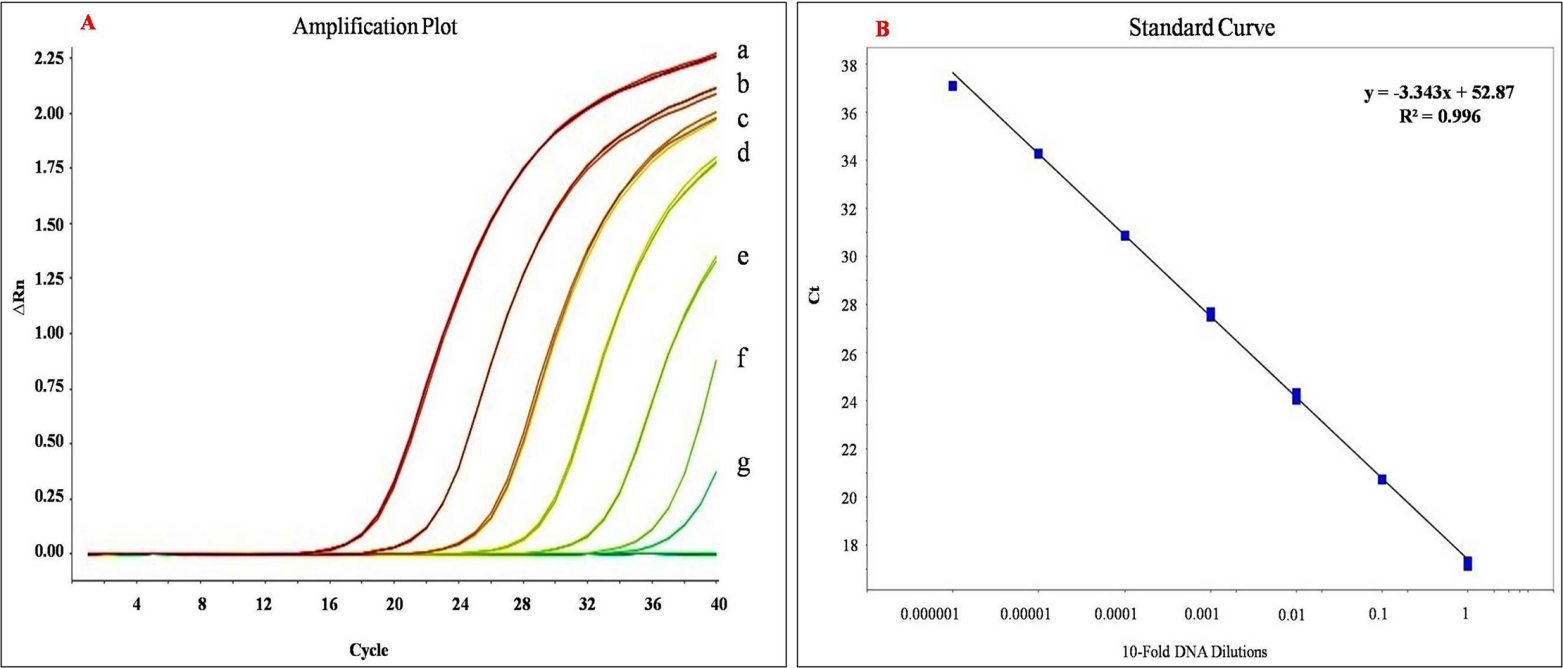
(A) qPCR Amplification plot generated by known concentration of *C*Las genomic DNA to check efficiency and sensitivity of TaqMan-qPCR with HLBas-F/Rn-HLBp primer probe pair, Line-a = 12.5 ng, Line-b = 1.25 ng, Line-c = 0.125 ng, Line-d = 0.0125 ng, Line-e = 1.25 pg, Line-f = 0.125 pg and Line-g = 12.5 fg template DNA. (B) Sensitivity of the primer-probe combination (HLBas-F/Rn-HLBp specific) for *C*Las detection using TaqMan qPCR assay. The standard curve established between log of DNA concentrations vs. cycle threshold (Ct) obtained using 10-fold serial dilution of total genomic DNA of Mosambi plants infected with *C*Las (initial concentration 12.5 ng/μl, final concentration 12.5 fg/μl).

The standardized TaqMan-qPCR assay consistently detected *C*Las in infected Mosambi plants and extracted DNA at concentrations ranging from 0.0125 pg to 12.5 x 10^3^ pg (Fig 3A). The assay showed standard fluorescence with exponential amplification of PCR amplicon and the standard curve was generated (Fig 3B). The association between Ct value and DNA quantities were robust with correlation coefficient (*R*^2^) of 0.996 for HLBas-F/Rn-P and 0.999 for COX-F/R-P (the latter used as internal control). The pathogen detection limit of standardized qPCR assay was ≤ 0.0125 pg of total DNA from HLB infected plant. Despite uneven distribution of *C*Las bacteria, we were able to detect abundant amount of *C*Las (low Ct value) after standardization of the protocols *viz*., the annealing temperature and extension time. Thus, the TaqMan-qPCR assay was used subsequently to test the *C*Las load, before and after the application of antimicrobial 2S albumin protein and Nano-ZnO either individually or in combination.

### The quantification of *C*Las genome copy number in plant tissues

**Fig 4:**
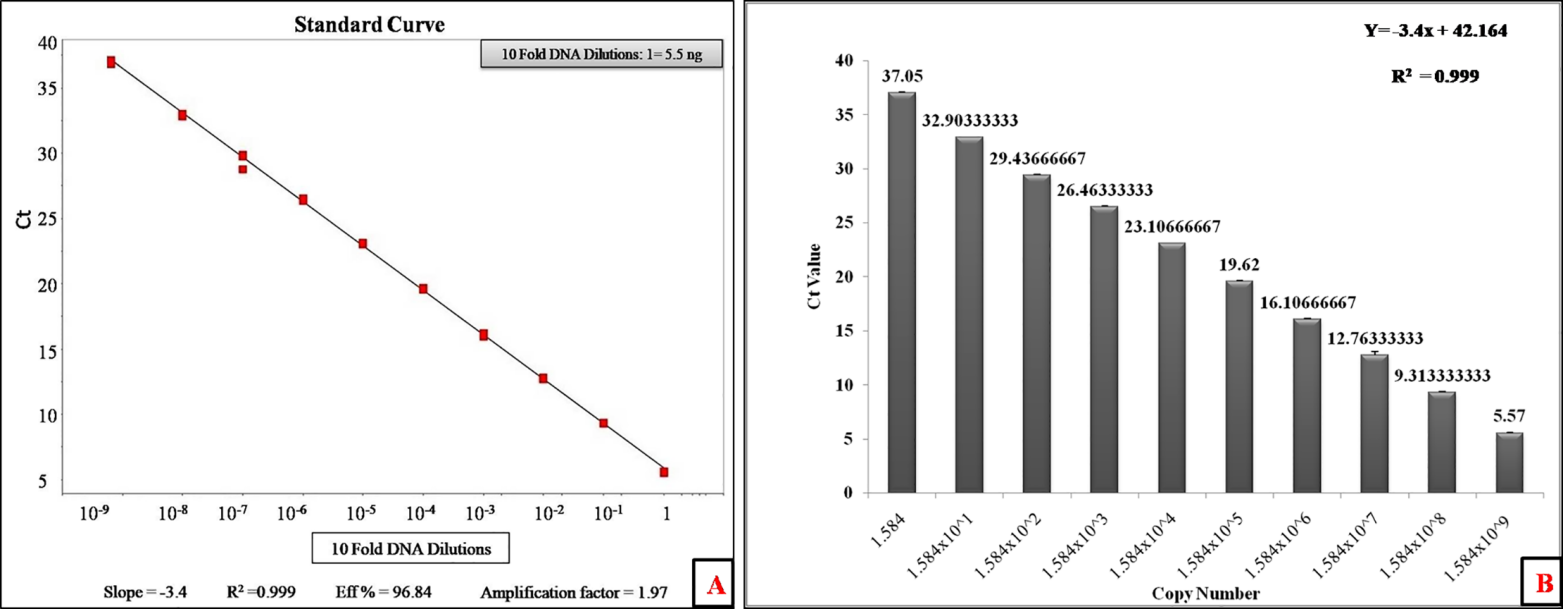
(A) The quantification of *C*Las genome copy number with standard curve. (B) The exponential relationship between copy number and Ct (Cycle Threshold) value.

The TaqMan-qPCR reactions were performed with known copy number and 10-fold serially diluted template DNA to determine exact relationship between Ct value and DNA copy number. The initial known template DNA concentration of 5.5 ng per reaction with copy number 1.584 x 10^9^ showed an average Ct value of 5.57. The exponential amplification of target amplicon was by at least 3.4 orders of magnitude (Fig 4A). The exponential relationship between DNA copy number and Ct value was also determined (Fig 4B). The quantification of *C*Las genome copy number after each treatment was done with the help of regression equation Y = -3.4x + 42.164 obtained from standard curve with regression coefficient R^2^ = 0.99 and Eff% = 96.9. The plant was considered as negative with *C*Las if the observed Ct value was >36 and was confirmed using HLB specific primers 3F/4R and OI1/OI2c.

### The effectiveness of 2S albumin protein and Nano-ZnO against *C*Las

All HLB affected Mosambi plants were analyzed for *C*Las titer individually after every treatment. The results showed a significant difference in *C*Las titer in control and treated plants with each treatment except Zinc nitrate alone (details given below). A substantial reduction in the *C*Las genome copy number was observed 30 (DAT) with each treatment administered individually. The *C*Las titer was also reduced after 60, 90,120 DAT, but the extent of reduction in *C*Las genome copy number was low 60 DAT.

### Effect of 2S albumin protein and Zinc nitrate on *C*Las

**Fig 5:**
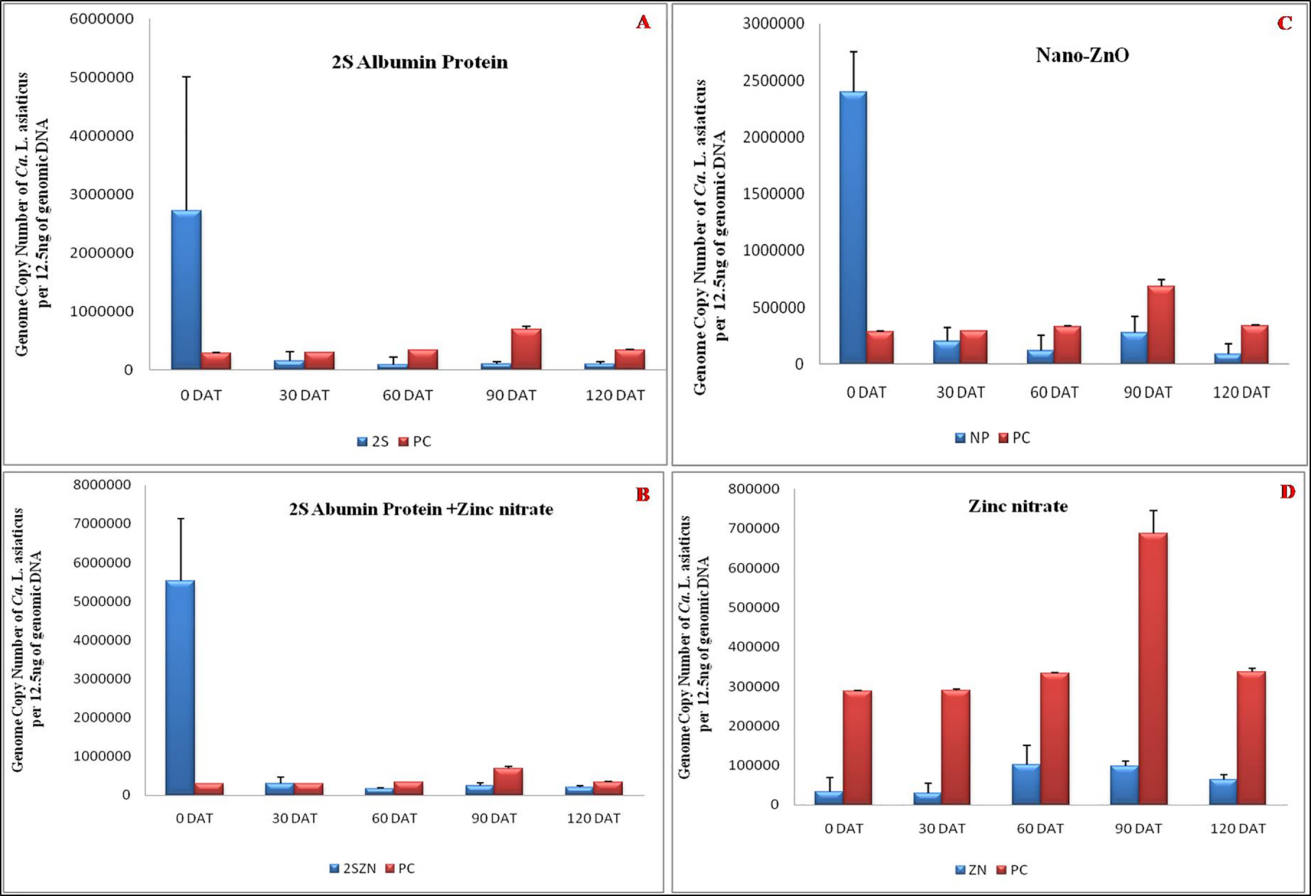
**The *C*Las titer of infected Mosambi seedling before and at 30, 60, 90,120 DAT of** (A) 2S albumin Protein, (B) 2S albumin Protein plus Zinc nitrate, (C) Nano-ZnO and (D) Zinc nitrate compared with untreated positive control (red colour bars indicates infected positive control without treatment whereas blue bars represent treated plants).

The plants (HLB positive) treated with PBS buffered 2S albumin protein were evaluated by TaqMan-qPCR for *C*Las titer every 30 DAT (Fig 5A). Prior to the treatment with 2S albumin protein, the estimated genome copy number of *C*Las in the plant was 2.72×10^6^ /12.5 ng of genomic DNA with Ct value 19.6, but after 30 DAT the mean Ct value increased to 23.91, indicating the decrease in the pathogen titer from 2.72×10^6^ /12.5 ng of genomic DNA to 1.55×10^5^. This means that 30 DAT, the genome copy number of *C*Las decreased by 2.5×10^6^. In other words, there is 17.47-fold decrement of *C*Las population in infected Mosambi plants 30 DAT (Table 1). Similarly, *C*Las load was also monitored 60, 90,120 DAT. The *C*Las genome copy number was decreased from 2.72×10^6^ /12.5 ng of genomic DNA to 9.15×10^4^, 9.9×10^4^, 9.5×10^4^ /12.5 ng of genomic DNA, respectively (Fig 5A). Overall, the estimated *C*Las population/titer is reduced by 17.47, 29.72, 27.44 and 28.56-fold after 30, 60, 90, and 120 DAT, respectively (Table 1). We also observed that, Zinc nitrate treatment alone did not have any effect on *C*Las titer (Fig 5D). Further, Zinc nitrate even when applied together with 2S albumin did not show any additive effect in reducing *C*Las titer (Fig 5B). This indicates that overall effect of 2S albumin protein either singly or coupled with Zinc nitrate was attributed to the 2S albumin protein alone.

### Effect of Nano-ZnO on *C*Las

Similar to 2S albumin protein, HLB affected plants were treated with Nano-ZnO (1,000 ppm) individually for evaluation of *C*Las titer every 30 DAT (Fig 5C). The effectiveness of Nano-ZnO was observed as parallel to the effects recorded with 2S albumin protein. Prior to treatment with Nano-ZnO, the average genome copy number of *C*Las was 2.3×10^6^ /12.5 ng of genomic DNA with a Ct value 19.8, but 30 DAT the average Ct value increased to 23.5, indicating the decrease of pathogen titer from 2.3×10^6^ /12.5 ng of genomic DNA to 2×10^5^. This means that 30 DAT with Nano-ZnO, the genome copy number of *C*Las decreased by 2.1×10^6^. This is 11.65-fold decrement of *C*Las titre in Mosambi plants 30 DAT. Similarly, the genome copy number was decreased by 19.56, 8.59, 26.72-fold when checked 60, 90, 120 DAT respectively (Table 3).

**Table 3:** Comparative effectiveness of 2S albumin protein and Nano-ZnO on growth and multiplication of *C*Las in planta.

DAT(Days after treatment)	Treatment and fold decrease					No treatment
	2S albumin	Nano-ZnO	2S albumin + Nano-ZnO	2S albumin + Zinc nitrate	Zinc nitrate	Positive control
30	17.47	11.65	27	18	1	1
60	29.72	19.56	41.69	33	0	0.8
90	27.44	8.59	23	23	0	0
120	28.56	26.72	34.16	28.18	0.5	0.8

Note: Negative value in fold change in Zinc nitrate treatment is due to increase in pathogen copy number. (The fold-change was determined by dividing the average of total pathogen titer before the treatment by the total titer of each at 30 DAT).

### Effect of 2S albumin plus Nano-ZnO

**Fig 6:**
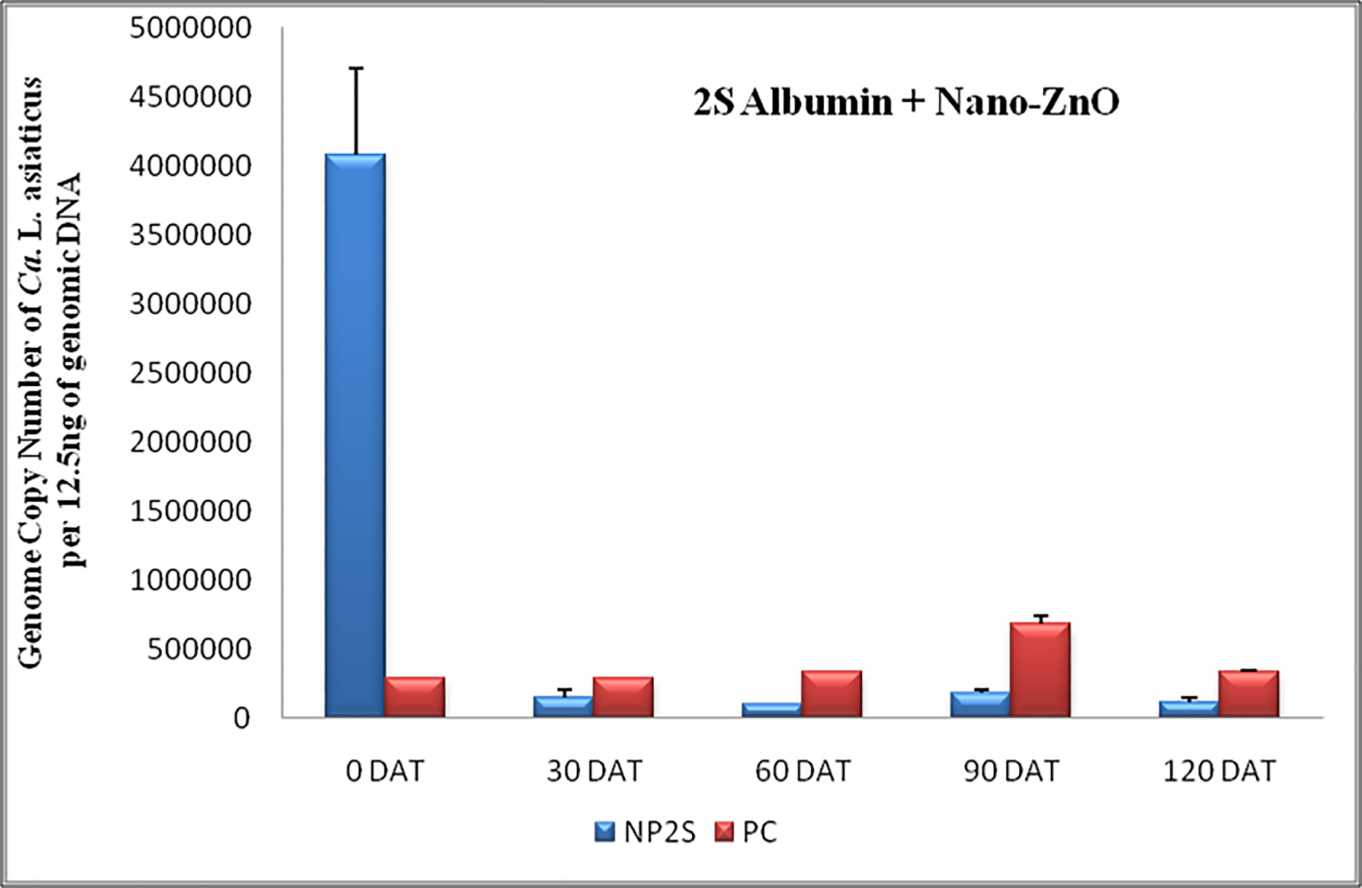
The *C*Las titer of infected Mosambi plants before the treatment and at 30, 60, 90, 120 DAT with 2S albumin proteins plus Nano-ZnO compared with control plants. (red color bars indicate without treatment whereas blue bars represent treated plants).

**Fig 7:**
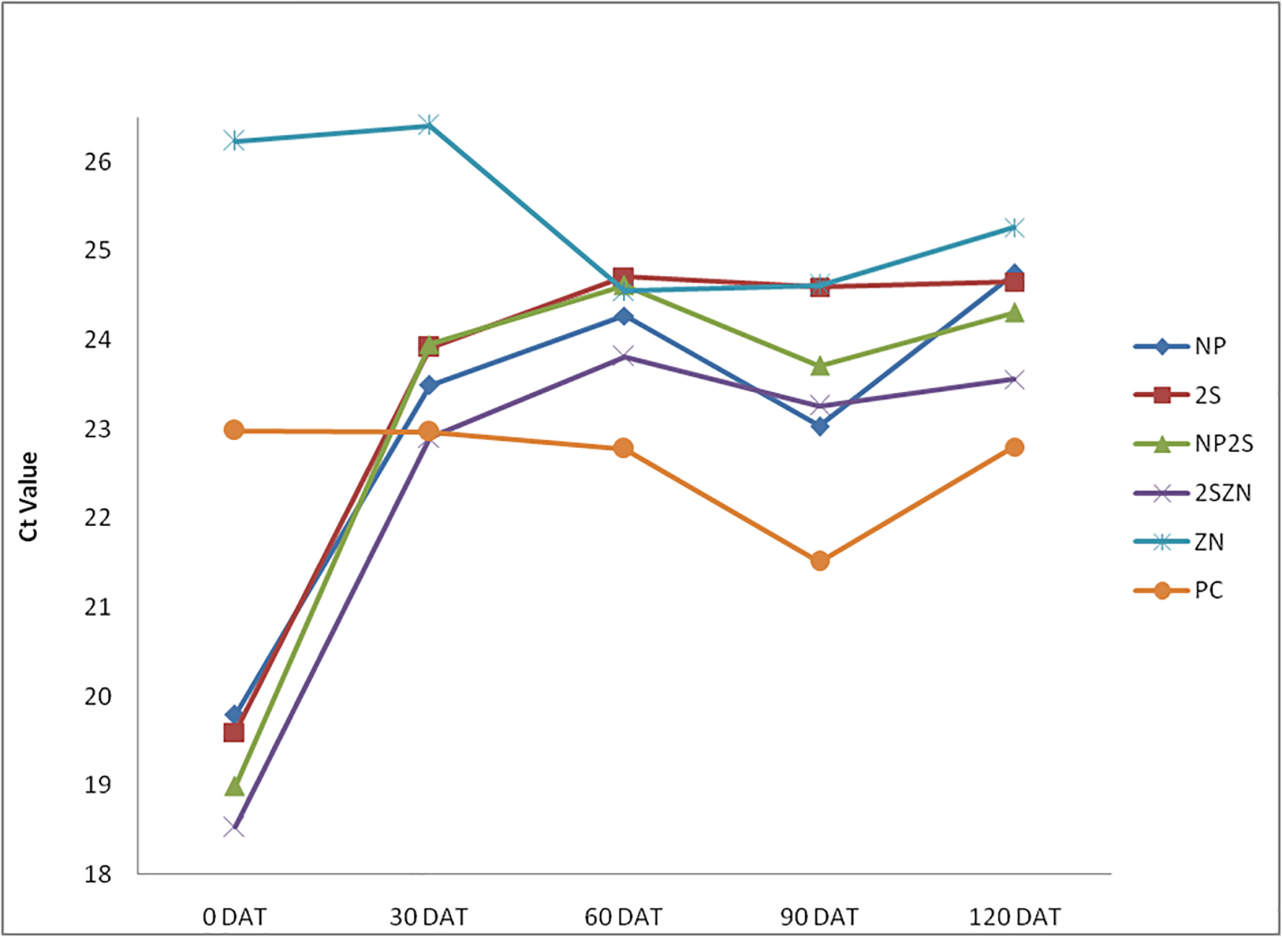
The effect of different treatment on HLB titer 30, 60, 90, and 120 DAT, compared with positive control (untreated seedlings). The treatments were: NP (Nano-ZnO), 2S (2S albumin protein alone), NP2S (Nano-ZnO plus 2S albumin proteins), 2SZN (2S albumin Protein plus Zinc nitrate), ZN (Zinc nitrate) and PC (Positive control only injected with PBS).

The major emphasis of our study was to determine the effects of 2S albumin protein and Nano-ZnO on *C*Las titer in infected citrus plant. In addition to the individual treatment, we also monitored the combined effect of both compounds at on *C*Las titer (Fig 6). The initial *C*Las load at 0 DAT was 4×10^6^/12.5 ng of genomic DNA, while at 30 DAT (2S albumin plus Nano-ZnO at the ratio of 1:1) the *C*Las population reduced to 1.5×10^5^. The rate of suppression of HLB titer is high in 2S albumin plus Nano-ZnO treatment as compared to individual treatments. This might be due to combined effect of both compounds on the growth and multiplication of *C*Las inside the phloem. It was observed that the bacterial titer reduced by 27, 41.69, 23 and 34.16-fold after 30, 60, 90 and 120 DAT respectively (Table 3). The comparative effectiveness of 2S albumin protein, Zinc nitrate, Nano-ZnO and the relevant controls are summarized (Fig 7).

## Discussion

HLB is economically the most serious and destructive disease of citrus and has severally affected the citrus industry as no other disease throughout the world. The rapid proliferation of *C*Las population in phloem tissue would deliver virulence factors and effector proteins into the phloem of HLB affected citrus plant causing malfunction of phloem tissue (*i*.*e*. cell death, necrosis and whole plant dieback) [4, 53]. The *C*Las infection also leads to reduction in plant vigour, flowering and fruit yield. Because of the knowledge gap in complete understanding of *C*Las and its pathogenesis, there is difficulty in controlling *C*Las infection progression and development of management strategies to mitigate the pathogen's infection progression. Since *C*Las pathogen resides and restricted to the phloem, there are significant challenges in development of effective antimicrobial strategies [30]. There are several reports, where certain antibiotics *i*.*e*. ampicillin, streptomycin may reduce the inoculums load of *C*Las [27], but owing to inconsistent effect, the commercial practice of these chemicals is very limited. Thus, major emphasis of this investigation was to explore the antimicrobial activity of the 2S albumin, a plant storage protein to induce microbial inhibition. The 2S albumin family protein, SiAMP2 from *Sesamum indicum* kernels was shown to possess antimicrobial properties [47]. The 2S Albumin protein of ~16 kDa, from Seeds of *Wrightia tinctoria* showed antibacterial activity against human pathogen *Morexalla catarrhalis* [54]. Thus, efforts were made to seek novel antimicrobials against *C*Las infection progression with different mechanisms of action.

Recently the 2S albumin protein has been characterized from the seeds of pumpkin (*Cucurbita maxima*) and Putranjiva (*Putranjiva roxburghii*), a heterodimeric protein of ~12 kDa, with RNase, DNase and antimicrobial activities [49]. Recent interest in the application of engineered Nano-ZnO formulation to improve the immunity of different agricultural crops against bacterial and fungal pathogens has gained increased momentum. There are reports of the effective antibacterial activity of Nano-ZnO against *E*. *coli*, *P*. *aeruginosa* and *S*. *aureus* [40, 55]. This inhibitory effect of Nano-ZnO is due to its ability to damage bacterial cell membrane and their consequent death [56]. Use of Nano-ZnO as an effective antibacterial agent to protect citrus industry from destructive HLB has been suggested [55]. In this study, we tried to combine the antibacterial attributes of 2S albumin protein and Nano-ZnO to treat *C*Las infection in Mosambi (*Citrus sinensis*) plants.

*C*Las titer was monitored during the experimental period using highly sensitive, specific and robust TaqMan-qPCR assay. Except Zinc nitrate treatment, inhibitory effect against *C*Las was observed with all four treatments. In the case of 2S albumin protein alone, the pathogen titer reduced gradually after each treatment. The reduction rate was very high initially at 94% of initial bacterial titer 30 DAT bacterium inoculums 30 DAT and 96.63%, 96.35%, 96.49% after 60, 90 and 120 days of treatment respectively. In other words, we found that when 2S albumin protein was applied individually, there was more than 28-fold reduction of *C*Las titer after 60 days of treatment and no change observed after 120 days as compared to control plants (Table 3). We also observed that Nano-ZnO treatment also suppressed *C*Las population. Overall effectiveness of Nano-ZnO was lower than 2S albumin protein as evidenced by their reduced copy number and pathogen titer (Fig 5C). However, treatment of 2S albumin protein in combination with Nano-ZnO showed the highest reduction of *C*Las titer 30 DAT and sustained titer reduction even upto120 DAT. A maximum of 41.69-fold decrease of bacterial titer after 60 days of treatment was recorded with the combined treatment at any given time (Figs 6, 7 and Table 3).

The present strategy of combining 2S albumin with Nano-ZnO treatment, were successful in reducing *C*Las infection progression in phloem of Mosambi plants. The reduction of *C*Las titer was observed in treated plants in comparison with untreated control plants (statistically significant, p < 0.05). The additive effect of 2S albumin and Nano-ZnO together with no observable adverse effect on the health of plants even ten months after trunk injection potentially could be used to mitigate the spread of CLas.

In summary, we have demonstrated for the first time that antimicrobial 2S albumin protein in combination with Nano-ZnO significantly reduced *C*Las titer in HLB affected Mosambi plants in comparison to HLB affected untreated control. The actual mechanism of action of 2S protein and the 2S protein plus the Nano-ZnO remain to be determined. However, the antimicrobial property of Nano-ZnO is primarily attributed to the release of significant amounts of Zn^2+^ ions locally due to large surface area to volume ratio of ultra-small size (<5 nm) particles [57]. Other possibilities include direct interaction of Nano-ZnO to *C*Las, resulting in disruption of the cell membrane and causing oxidative stress [58], as well as binding interaction of Nano-ZnO to albumin [59, 60]. Additionally, NAC coated Nano-ZnO might have contributed as an efficient delivery of the 2S antimicrobial protein to the phloem for targeting *C*Las, significantly suppressing its population.

## Conclusion

To the best of our knowledge, this is the first report which demonstrate the *in planta* efficacy of two antimicrobial compounds against *C*Las *viz*. 2S albumin (a plant based protein), Nano-Zinc Oxide (Nano-ZnO) and their combinations. Application of 2S albumin and Nano-ZnO formulation alone or in various combinations showed marked reduction in *C*Las titers in affected plants as compared to untreated plants where the pathogen titers were approximately stable throughout the time course under screen house conditions. Among all five treatments, 2S albumin-Nano-ZnO formulation performed best and showed significant reduction of *C*Las titer. It also confirms the potency of 2S albumin-Nano-ZnO formulation as an antimicrobial treatment for suppressing *C*Las *in planta* and could potentially be developed as a novel anti *C*Las therapeutics to mitigate the HLB severity affecting the citrus industry worldwide.

## Supporting information

S1 Fig (a) and (b) HRTEM images of the synthesized Nano-ZnO. (c) particle size distribution histogram calculated from HRTEM studies via processing 7 images and total number of 89 particles, the average size was found to be 4.1 nm ± 0.7 nm. (d) DLS histogram of particle size distribution, the average hydrodynamic diameter of Nano-ZnO is calculated to be about 20.5 ± 6.7 nm. (e) Zeta-potential (ζ) data, the ζ value at pH 7.50 is estimated to be -20.0 mV ± 7.1 mV, confirming negative surface charge of Nano-ZnO
(TIF)

S2 Fig Phytotoxicity assessment of Nano-ZnO was carried out using vinca (vincire) plants (obtained from local Home Depot store), the images were taken after 72 hours of foliar application. **(**a) DI water was non-phytotoxic as expected. (b) Nano-ZnO at 300 μg/ml, (c) Nano-ZnO at 600 μg/ml, and (d) Nano-ZnO at 1,000 μg/ml were found to be non-phytotoxic. (e) Zinc nitrate at 300 μg/ml was found to be non-phytotoxic, however, (f) Zinc nitrate at 600 μg/ml was found to be minimal phytotoxic, and (g) Zinc nitrate at 1,000 μg/ml was found to be moderately phytotoxic. (h) Copper nitrate was chosen to test the phytotoxicity study conditions and it was found to be severely phytotoxic at 1,000 μg/ml.
(TIF)

S1 File Phytotoxicity rating scale was assessed via Vinca plants treated with different concentrations of Nano-ZnO, zinc nitrate, DI water and copper nitrate. The assessment was done visually based on size and number of burns on plants after 72 hours of foliar application. Vinca (*vincire*) plant phytotoxicity rating on a scale of “-” non-phytotoxic, “+” minimal phytotoxic, “++” moderate phytotoxic, and (+++) heavy phytotoxic.
(DOCX)
