# Supplementary figures and images for: Antimicrobial nano-zinc oxide-2S albumin protein formulation significantly inhibits growth of “Candidatus Liberibacter asiaticus” in planta
Source: PLoS One. 2018 Oct 10;13(10):e0204702. doi: 10.1371/journal.pone.0204702 (PMC6179220; doi:10.1371/journal.pone.0204702)

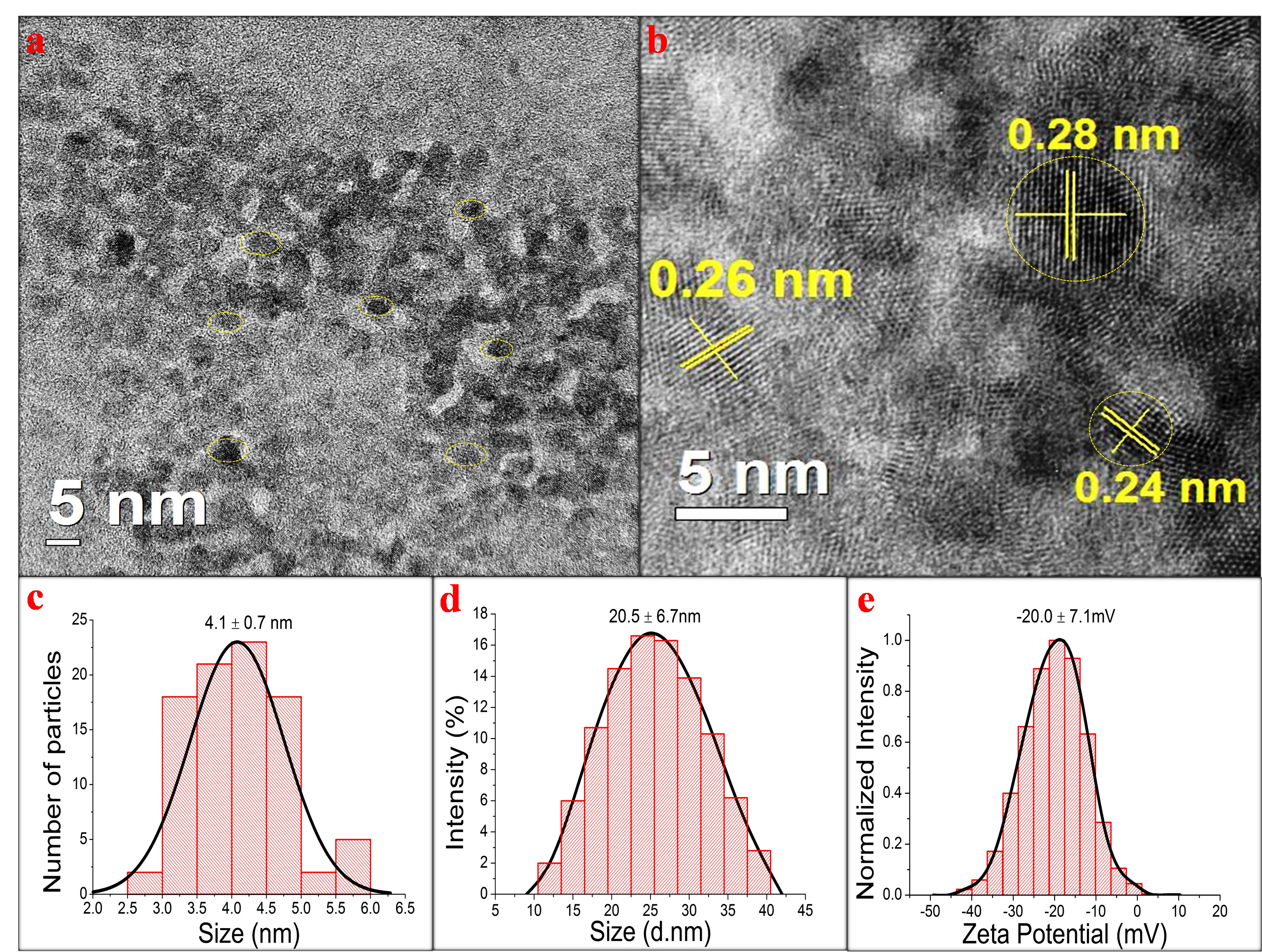

Supplement: S1 Fig — (a) and (b) HRTEM images of the synthesized Nano-ZnO. (c) particle size distribution histogram calculated from HRTEM studies via processing 7 images and total number of 89 particles, the average size was found to be 4.1 nm ± 0.7 nm. (d) DLS histogram of particle size distribution, the average hydrodynamic diameter of Nano-ZnO is calculated to be about 20.5 ± 6.7 nm. (e) Zeta-potential (ζ) data, the ζ value at pH 7.50 is estimated to be -20.0 mV ± 7.1 mV, confirming negative surface charge of Nano-ZnO (TIF) [file pone.0204702.s001.tif]

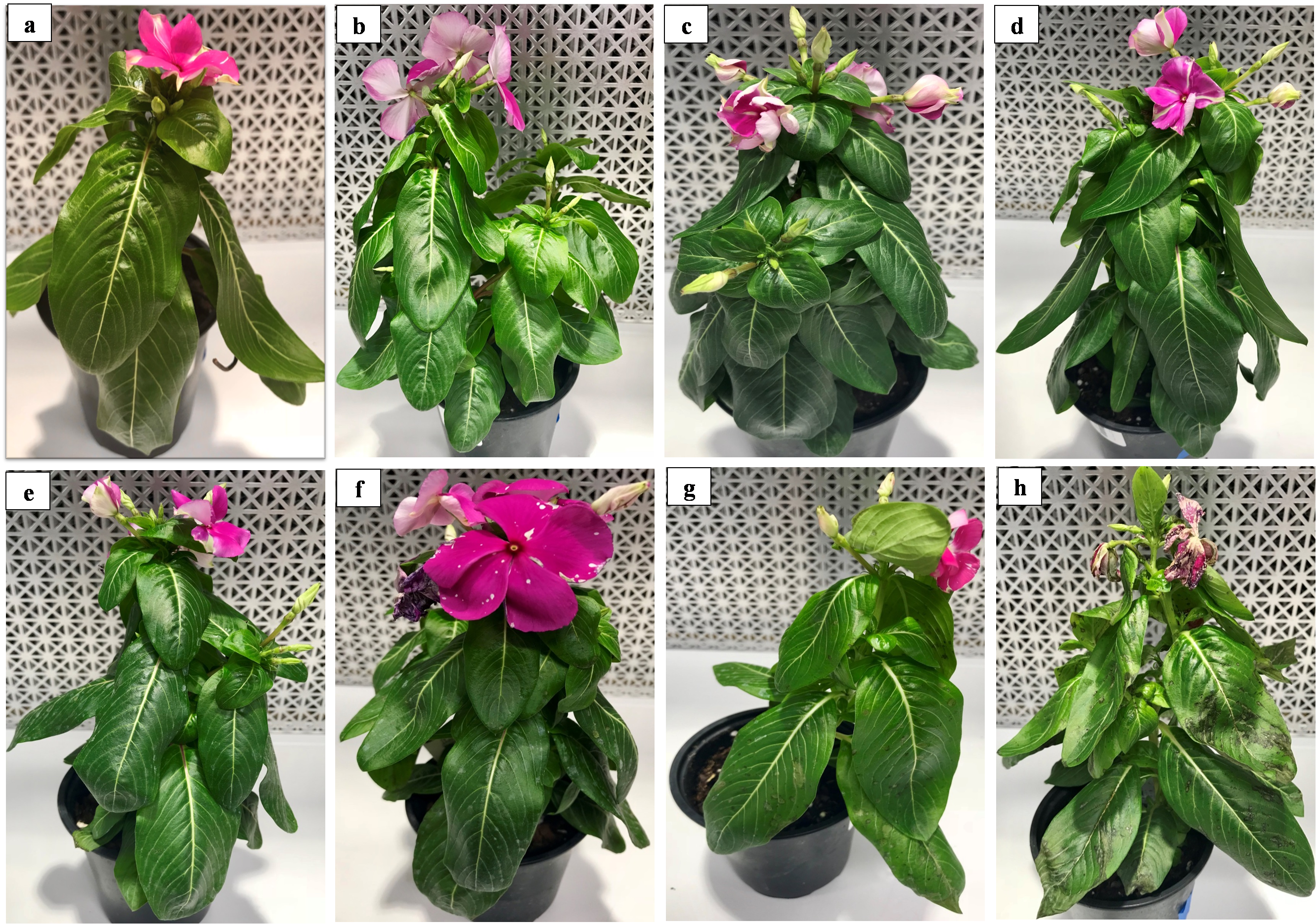

Supplement: S2 Fig — (a) DI water was non-phytotoxic as expected. (b) Nano-ZnO at 300 μg/ml, (c) Nano-ZnO at 600 μg/ml, and (d) Nano-ZnO at 1,000 μg/ml were found to be non-phytotoxic. (e) Zinc nitrate at 300 μg/ml was found to be non-phytotoxic, however, (f) Zinc nitrate at 600 μg/ml was found to be minimal phytotoxic, and (g) Zinc nitrate at 1,000 μg/ml was found to be moderately phytotoxic. (h) Copper nitrate was chosen to test the phytotoxicity study conditions and it was found to be severely phytotoxic at 1,000 μg/ml. (TIF) [file pone.0204702.s002.tif]
