# Supplementary material for: Antimicrobial nano-zinc oxide-2S albumin protein formulation significantly inhibits growth of “Candidatus Liberibacter asiaticus” in planta
Source: PLoS One. 2018 Oct 10;13(10):e0204702. doi: 10.1371/journal.pone.0204702 (PMC6179220; doi:10.1371/journal.pone.0204702)
Supplement: S1 File — The assessment was done visually based on size and number of burns on plants after 72 hours of foliar application. Vinca (vincire) plant phytotoxicity rating on a scale of “-” non-phytotoxic, “+” minimal phytotoxic, “++” moderate phytotoxic, and (+++) heavy phytotoxic. (DOCX) [file pone.0204702.s003.docx]

**High Resolution Transmission Electron Microscopy (HRTEM)**

A dilute nano-ZnO solution (25 ppm metallic Zn) was sonicated for 30 minutes using Elmasonic S30H sonic bath. 5 µL of the sonicated solution was drop-casted on a TEM grid (Electron Microscopy Sciences, catalogue # CF300-AU-UL) and air-dried overnight. The measurements were carried out via FEI Tecnai F30 TEM instrument. For generating a histogram of particle size distribution, we have measured size of about 200 particles from several HRTEM images using Gatan Microscopy Suite (GMS) 3 software. Fast Fourier Transform (FFT) analysis was done on HRTEM images to understand the crystalline phage of nano-ZnO. The lattice spacing distance (d-values, A°) obtained through FFT analysis were compared to Powder Diffraction Files (PDF) of various possible Zn related compounds reported to International Center for Diffraction Data (ICDD).

**Dynamic Light Scattering (DLS) and Zeta Potential**

DLS and zeta potential measurements were carried out at 25°C in DI water using Malvern Zetaziser ZS90. DLS measurements were performed using disposable polystyrene cuvette (Malvern Company; product# DTS0012) and zeta potential measurements were performed using folded capillary zeta cells (Malvern; product# DTS1070). Nano-ZnO solution at 1000 ppm metallic Zinc concentration was vortex mixed and sonicated for around 5 minutes in order to fully disperse particles.

**Table.** Phytotoxicity rating scale was assessed via Vinca plants treated with different concentrations of Nano-ZnO, zinc nitrate, DI water and copper nitrate. The assessment was done visually based on size and number of burns on plants after 72 hours of foliar application. Vinca (*vincire*) plant phytotoxicity rating on a scale of “-” non-phytotoxic, “+” minimal phytotoxic, “++” moderate phytotoxic, and (+++) heavy phytotoxic.

| **Materials tested** | **Metal concentration**  **(µg/ml)** | **Phytotoxicity rating** |
| --- | --- | --- |
| **DI water** | NA | - |
| **Nano-ZnO** | 300 | - |
|  | 600 | - |
|  | 1000 | - |
| **Zinc nitrate** | 300 | - |
|  | 600 | + |
|  | 1000 | ++ |
| **Copper nitrate** | 1000 | +++ |
